# Identification and distribution of *Brachyspira* species in feces from finishing pigs in Argentina

**DOI:** 10.14202/vetworld.2021.607-613

**Published:** 2021-03-10

**Authors:** Alicia Carranza, Julián Parada, Pablo Tamiozzo, Malena Flores León, Pablo Camacho, Gabriel Di Cola, Enrique Corona-Barrera, Arnaldo Ambrogi, Gustavo Zielinski

**Affiliations:** 1 Department of Animal Pathology, Faculty of Agronomy and Veterinary, National University of Rio Cuarto, Río Cuarto, Argentina; 2 National Scientific and Technical Research Council (CONICET), Córdoba, Argentina; 3 Faculty of Veterinary Medicine and Animal Science, Autonomous University of Tamaulipas, Victoria, México; 4 National Agricultural Technology Institute (INTA), Marcos Juárez, Córdoba, Argentina

**Keywords:** *Brachyspira* species, diagnosis, herd prevalence, intestinal spirochetes, swine

## Abstract

**Background and Aim:**

*Brachyspira* are Gram-negative, aerotolerant spirochetes that colonize the large intestine of various species of domestic animals and humans. The aim of this study was to determine the presence and distribution of different species of Brachyspira presents in feces from finishing pigs in Argentina.

**Materials and Methods:**

Fecal samples (n=1550) were collected from finishing pigs in 53 farms of the most important swine production areas of Argentina, and *Brachyspiras* species were identified by bacteriological and molecular methods.

**Results:**

The regional prevalence of *Brachyspira* spp. was at the level of 75.5% (confidence interval 95%, 62.9-87.9), and it was lower among those farms with >1001 sows. One hundred and twenty-eight isolates of *Brachyspira* were properly identified and the species found were: *Brachyspira hyodysenteriae*, *Brachyspira pilosicoli*, *Brachyspira innocens*, and *Brachyspira murdochii*. *B. hyodysenteriae* and *B. pilosicoli* had low prevalence (1.9% and 7.5%, respectively), *B. innocens* was isolated from 34% of the farms and *B. murdochii* was found in 39.6%.

**Conclusion:**

The present study provides epidemiological data about herd prevalence of the different *Brachyspira* species in Argentina, showing that the prevalence figure seems to be higher than that reported in other countries.

## Introduction

*Brachyspira* are Gram-negative, aerotolerant spirochetes that colonize the large intestine of various species of domestic animals and humans. *Brachyspira hyodysenteriae* has been recognized as the etiological agent of swine dysentery, a swine disease characterized by a severe muco-hemorrhagic colitis in growing pigs, and *Brachyspira pilosicoli* is responsible for a condition known as porcine colonic spirochetosis, which has a negative impact on pig production as a consequence of the muco-catarrhal colitis, green or brown diarrhea, and the poor performance of fattening pigs [1]. Other known species of the genus *Brachyspira* are, *Brachyspira innocens*, *Brachyspira murdochii*, and *Brachyspira intermedia* which are also found in pigs colon; these species are also responsible for mild colitis in pigs except *B. innocens* that is regarded as non-pathogenic. However, the previous reports on clinical cases and experimental studies [2,3] have found that one single species of *Brachyspira* or mixed infection, that is, various species of *Brachyspira* in a host could cause mild degrees of colitis. Other reports showed *B. innocens*, *B. murdochii*, and *B. intermedia* individually associated with pathological colitis [4], or in mixed infections with other infectious agents [5]. Recently, *Brachyspira hampsonii* has been recognized as a new species of *Brachyspira* that is pathogenic as it has been isolated from clinical cases of swine dysentery in Canada [6].

Isolation and identification of *Brachyspira* spp. by bacteriology are not a straightforward approach, because of their slow growth and the difficulty of getting isolates in pure culture. At present, different molecular methods are available for detection and identification of *Brachyspira* species from fecal samples [7,8], or from bacteriological cultures [9], but the protocols need to be updated continuously [10]. One of the most useful genes to assess the diagnosis of *Brachyspira* spp. is the NADH oxidase (*nox)*, a relatively conserved gene amongst *Brachyspira* spp., which allows differentiation and proper identification of the different species [9]. In Latin America, limited studies were carried out concerning the presence of different species of *Brachyspira*. In Brazil, some regional studies identified *B. hyodysenteriae* and *B. pilosicoli* in pigs [11,12]. In Mexico, strongly and weakly hemolytic spirochetes were identified in 22% of pig farms [13]. In Argentina, although both pathogenic and non-pathogenic *Brachyspira* spp. have been identified by different diagnostic methods, very few epidemiological studies have been conducted, and thus little is known about the presence and distribution of the different *Brachyspira* species [14].

The aim of this study was to determine the presence and distribution of different species of *Brachyspira* present in feces from finishing pigs in Argentina.

## Materials and Methods

### Ethical approval

The study was approved by “Comité de Ética de la Investigación” (RR 852/11) of the National University of Río Cuarto (UNRC). All animal procedures carried out in our study were performed in accordance with international regulations.

### Study period and location

In order to determine the prevalence of infection in the Pampean region of Argentina, the main pig producing area in the country, 53 commercial farrow-to-finish pig farms were sampled between October 2011 and March 2013.

### Farms, animals, and samples

According to the 2012 Integrated System for Animal Health Management, provided by the National Animal Health, Food Safety and Quality Service of Argentina, at the moment of the present study, the number of confinement pig farms with more than 200 sows in the region was 322, with a total figure of 153,350 sows. Most farms (84%, 270 farms) were located in the provinces of Buenos Aires (37%), Cordoba (24%), Santa Fe (16%), and Entre Ríos (7%). The number of farms necessary to assess the prevalence of *Brachyspira* spp. positive farms in the region was calculated according to Thrusfield [15], considering an expected prevalence of 50%, with 95% of confidence, and a precision of 13%. Then, a stratified sampling method was designed according to the number of farms in each province.

The number of fecal samples to be collected was n=30 as to detect the presence of at least one positive sample for intestinal spirochaetes [15]. Samples were collected from pigs of 22 weeks of age, with or without diarrhea and taken directly from the rectum of pigs by manual stimulation, placed in polyethylene bags and put at 4°C until reaching the laboratory for processing, which was achieved no longer than 48 h after collection. A total of 1550 fecal samples were collected.

A farm was considered positive when *Brachyspira* spp. growth characteristic on selective culture plates was confirmed by the observation of Gram-negative stained spirochaetes under optical microscopy.

### Bacteriology and biochemical identification

The feces sample were plated onto *Brachyspira* selective medium (BSM) made up of Columbia Agar (Oxoid Ltd., Hants, UK) supplemented with 7.0% horse blood and the antibiotics colistin (25 mg/mL), vancomycin (25 mg/mL), and spectinomycin (400 mg/mL) (Rosco Diagnostica, Taastrup, Denmark). Inoculated plates were incubated at 42°C for 7 days in anaerobic jars, using AnaeroGen GasPak system (Oxoid Ltd., Hants, UK). After the incubation, smears were made from positive culture plates that showed the growing characteristic of *Brachyspira* (strongly or weakly hemolytic). *Brachyspira* isolation was confirmed by observation of negative Gram-stained spirochetes using light microscopy examinations. Prime isolates were subcultured on BSM to obtain pure cultures. *Brachyspira* isolates were identified by biochemical testing (Rosco Diagnostica, Taastrup, Denmark) for the preliminary identification of species according to a method previously described [16]. Pure isolates were at −70°C for further purposes.

### Identification by polymerase chain reaction (PCR)

DNA from isolates was extracted using an organic extraction method (DNAzol, Invitrogen, USA). Two PCR approaches were used for species-specific identification of the *Brachyspira* isolates: (i) Duplex PCR for the identification of *B. pilosicoli* and *B. hyodysenteriae* [7] and (ii) for any other *Brachyspira* species restriction fragment length polymorphism-PCR (RFLP-PCR) [9,17], with some modifications. For the RFLP-PCR, the products were digested with *Dpn* II and *Scf I* and the fragments were separated on a 3% agarose gel by electrophoresis and stained with ethidium bromide. The standardization of the PCR testing was done using DNA from reference strains of *B. hyodysenteriae*, *B. pilosicoli*, *B. murdochii* y, and *B. innocens* provided by Dr. Enrique Corona-Barrera (Mexico).

### Identification by sequencing

Sequencing was carried out particularly on isolates for which biochemical identification was not clear, so the species identification was done by *nox* gene PCR amplification followed by sequencing using primers as described previously [18]. Briefly, the PCR products were purified by the commercial kit Puriprep S (Inbio Highway, Tandil, Argentina), quantified (NanoDrop, ND 1000, Thermo Fisher Scientific, USA), and sequenced (ABI PRISM® 3130xl Genetic Analyzer, Applied Biosystem, CA, USA). The sequences were edited using BioEdit, aligned with ClustalW and compared with GenBank database using BLAST.

### Statistical analysis

*Brachyspira* prevalence was calculated by dividing the number of positive farms by the total number of sampled farms in a region or province. The prevalence of a particular *Brachyspira* species was calculated by dividing the number of positive farms for that particular species by the total number of sampled farms. Statistical analysis, including interquartile range (IQR), Pearson’s Chi-squared test, confidence interval (CI), and standard deviation (SD), was carried out using Epidat software version 3.1 (Xunta de Galicia, OPS-WHO, Spain).

## Results

The number of herds necessary to estimate the prevalence was n=49. In addition, four farms of 150 sows located in the area of the study were also included as they sent samples to the diagnostic laboratory at university, giving them a total of 53 farms in the study. Farms with more than 200 sows represented 15.2% of the total of farms, for which a figure of 40,585 sows was recorded. The mean of herd size in this study was 500 sows (IQR=300-1000).

The regional prevalence of herds infected with different *Brachyspira* species was 75.5% (CI 95%, 62.9-87.9); in other words, 40 of the 53 sampled farms were culture positive for *Brachyspira*. The highest occurrence of *Brachyspira* spp. was observed in the province of Córdoba. Many species of intestinal spirochetes were found in that region (Table-1). In the provinces of Buenos Aires, Córdoba and Santa Fe only *B. pilosicoli* was found, and in Córdoba Province, *B. hyodysenteriae* was also detected (Table-2).

**Table-1:** Distribution of farms, samples taken, and prevalence of herds positive to *Brachyspira* spp. by province, positive samples for each diagnostic test, and frequency of *Brachyspira* spp. shedding pigs within-herd.

Province	Samples		Positive sample/test					Provincial prevalence (%)	Within-herd frequency (%)
	
	Farms	Fecal samples	Biochemical test	PCR	Sequencing	Brachyspira nontypeable	Positive farm		
Buenos Aires	17	504	3	9	29	63	10	58.8	19.0
Córdoba	22	637	29	5	28	68	20	91	21.8
Santa Fé	8	229	2	4	12	20	5	62.5	16.1
Entre Ríos	2	60	-	-	-	10	2	100	16.6
Other	4	120	2	4	1	5	3	75	10
Total	53	1550	36	22	70	166	40	75.5	18.9 (Average)

PCR=Polymerase chain reaction

**Table-2:** Number of isolates of each identified *Brachyspira* species, number of positive farms, number of farms with only one or more than one *Brachyspira* species and regional prevalence of each identified *Brachyspira* species and distribution of the number of farms infected with each species in the different provinces.

*Brachyspira* species	Isolates	Positive farms	Farms with one *Brachyspira* species	Farms with more than one *Brachyspira* species	Prevalence of positive farms (%)	Geographical distribution			

						Buenos Aires	Córdoba	Santa Fé	Other
*Brachyspira hyodysenteriae*	5	1	0	1	1.9	-	1	-	-
*Brachyspira pilosicoli*	7	4	1	3	7.5	1	2	1	-
*Brachyspira innocens*	49	18	10	8	34.0	7	9	1	1
*Brachyspira murdochii*	67	21	12	9	39.6	6	8	5	2

Farms were categorized in quartiles (C1-C4) according to the number of sows, and the proportion of positive herds for each quartile was calculated (Table-3). No statistical difference between herd size and presence of *Brachyspira* was found. The prevalence of *Brachyspira* spp. was lower (37%) into the upper quartile (C4), among the larger farms (>1001 sows) (Chi-square Pearson=7.36, p=0.0613).

**Table-3:** Categorization of farms in quartiles according to herd breeding size, number of *Brachyspira* spp. positive farms, and percentage of positive farms in each category.

Category	n	Positives	% farms +
<300 sows (C1)	18	15	81.30
301-500 sows (C2)	11	9	81.80
501-1000 sows (C3)	16	13	81.25
>1001 sows (C4)	8	3	37.50

Characteristic growth of intestinal spirochetes on cultured plates was confirmed by Gram-stained smears, followed by microscopy. Isolation of *Brachyspira* on bacteriological culture was achieved on 19% (290/1550) of the cultured samples. The mean of *Brachyspira* positive cultures per farm was 18.9% (SD=17.3, range 0-62.1), and the highest figure of positive culture was 50-62% observed in four farms with a herd size between 680 and 1200 sows.

One hundred and 28 *Brachyspira* isolates were properly identified by the different testing procedures; the *Brachyspira* species found in this study were *B. hyodysenteriae, B. pilosicoli*, *B. innocens*, and *B. murdochii* (Table-2). Although samples (n=30) from eight farms were recorded as *Brachyspira* culture positive, these isolates were not possible to identify by the molecular techniques. It was also observed that more than one species of *Brachyspira* was found in 22.5% (9/40) of the farms.

*B. pilosicoli* was isolated from farms of Buenos Aires (n=1), Córdoba (n=2), and Santa Fe (n=1). In some farms, *B. pilosicoli* was found alone in 16.7% (5/30) of the samples, whereas in other farms, this species was found together with *B. innocens* and *B. murdochii* in up to 23.3% and 46.6% of the samples, respectively.

Indole-negative *B. hyodysenteriae* was identified in one herd from Córdoba, in which 16.7% (5/30) of fecal samples were culture positive.

The most frequent *Brachyspira* species found were *B. innocens* and *B. murdochii*. *B. innocens* was found in 45% (24/53) of the farms and in ten farms, it was identified as the only *Brachyspira* species ranging from 3% to 30% of the positive samples, this species was also found in 8 (12.2%) of the farms together with *B. hyodysenteriae* (n=1), *B. pilosicoli* (n=2), and *B. murdochii* (n=8). The most frequent *Brachyspira* species found in this study was *B. murdochii*, as it was present in 53% (28/53) of the *Brachyspira* culture positive farms. It was also recorded that some of the farms (n=4) had mixed *Brachyspira* infections, as found in four different farms where at least one sample of each farm had more than one *Brachyspira* species.

## Discussion

This study was conducted on pig farms located in the most important swine production areas of Argentina, where there was no previous data recorded on the prevalence of *Brachyspira*. Four additional farms located in neighboring provinces with small number of sows were also included in the study.

*Brachyspira* was isolated from feces of pigs of all the provinces included in this study, which gave an isolation rate of 76% (40/53) of the samples taken from 53 farms with more than 200 sows. The prevalence of *Brachyspira* in pig farms has been reported in other countries. For instance, in 2000, it was found that 50% of the farms in Denmark were positive to isolation of intestinal spirochetes as *Brachyspira* was found in 79 pig farms; moreover, in that study, other porcine enteropathogens were also reported [19]. In Mexico, out of a total of 73 farms from pig production areas, 16 (21.9%) were culture positive for intestinal spirochetes showing strong and weak hemolysis on blood agar plates [13]. However, the prevalence of *Brachyspira* positive farms in Argentina seems to be higher than that of Denmark and Mexico, whereas, in a study in Poland including finishing pigs from 20 farms, the prevalence of *Brachyspira* spp. was higher than 85% [20].

In other studies, intestinal spirochetes have been found in 63.2% (24/38) of pig farms in the state of Rio Grande do Sul (Brazil) and a remarkable prevalence of 100% was found in 22 farms with no use of antibiotics in the feed [11]. The relationship between the use of in-feed antibiotics and *Brachyspira* prevalence has been suggested previously [21,22]. Thereby, the use of antibiotics in the feed may be related to the high isolation rate found in our study, particularly in smaller farms (<300 sows), where the use of antibiotics was less frequent (data not shown). The age of pigs at the time of sampling in this study may also influence the high prevalence found, as it has also been reported in a recent study [20].

Although no statistical association was found between herd size and isolation of *Brachyspira* spp., the prevalence of *Brachyspira* was lower in farms with >1001 sows (C4). That could be due to more effective biosecurity measures and strategic programs on the use of antibiotics coupled with more technology applied to production.

In this study, a total of 290 samples were positive isolated and out of those, 128 isolates (44%) were properly identified at the species level (Tables-1 and 2). In some studies, bacteriological culture has shown higher sensitivity for detecting *B. hyodysenteriae* and *B. pilosicoli* as compared to PCR [8] or other available diagnostic tests [23]. In our study, samples were considered positive when the characteristic hemolysis on culture plates was seem and confirmed by observing Gram-negative spirochetes on microscopic preparations. However, for some samples, it was not possible to identify them fully at species level. *Brachyspira* is regarded as a fastidious microorganism, which is a feature that reduces the success of identification of prime isolates as the growth sometimes is so poor that its replication ends up in no further growth on subcultured plates, so the amount of growth might not be sufficient in the first instance. In other cases, it was not possible to get pure culture after repeated subculturing.

Several studies have shown drawbacks on the identification of *Brachyspira* isolates. A study reported that out of 876 samples, only 67 intestinal spirochaetes isolates were obtained and just a few were identified but not so accurate due to variations on biochemical testing [13]. It has been pointed out that as there are known variations and discrepancies on the identification of *Brachyspira* species using biochemical tests, genotype identification is preferred and absolutely necessary for the proper identification of *Brachyspira* species [9]. Other studies have also reported issues on the identification of *Brachyspira* isolates by PCR, as there was trouble on 15% of the isolates in a study on *Brachyspira* from swine [4]. The issues on the identification of *Brachyspira* isolates could be due to genetic alterations on the bacterial genome, which may lead to no amplification of genetic material or no detection by the primers used in the PCR test. It is also known that more than one species of *Brachyspira* could be present in the same sample, so this might interfere with the punctual identification of the species by PCR techniques.

The combination of bacteriological and molecular techniques has been used in several studies to address the difficulty of getting mixed results for the identification of samples at the species level [8], or to achieve proper detection of *Brachyspira* in mixed culture samples, or to identify a new species, such as *Brachyspira suanatina* which showed the same phenotype as *B. hyodysenteriae* [24]. Phenotypic variations are frequent in strains of *B. hyodysenteriae*; weakly hemolytic strains or negative indole strains may be present in a low percentage, as identified in the present study, which suggests that they must be confirmed by molecular techniques or sequencing [25-27]. But also, as Hampson *et al*. [28] have proposed, the diagnostic methodology needs to be reviewed regularly but should include both culture and molecular techniques. In our study, the combination of diagnostic testing allowed us the identification of more isolates at species level, which was more encouraging for our research.

The novel *Brachyspira* species, *B. hampsonii* and *B. suanatina*, were not identified by sequencing among the strong hemolytic isolates in our study. This is an important outcome as those species have been recognized as emergent species in clinical cases of swine dysentery [6,24].

In our study, *B. hyodysenteriae* and *B. pilosicoli* were found in low prevalence (1.9% and 7.5%, respectively) at the Pampean region of Argentina, even lower than that described in other studies (8.3% and 16.6%, respectively) [14]. This may be related to the sampling strategy in the present study, where feces samples were taken from fattening pigs with and without diarrhea. In fact, isolation of *B. hyodysenteriae* from herds with clinical signs of swine dysentery was previously reported (data not shown). In other countries, *B. hyodysenteriae* was identified in 30.4% (24/79) of the isolates from pigs with diarrhea, while other *Brachyspira* species were isolated at a lower rate [4]. However, in a study from 600 samples of 20 farms with the previous infection history for *B. hyodysenteriae*, 24% of the samples were identified among this species, while 45% corresponded to *B. innocens* and a lower percentage for *B. murdochii* and *B. pilosicoli*, 13, 5, and 9.4%, respectively [20].

The prevalence of *B. pilosicoli* in our study is similar to that reported in Brazil, where the isolation rate of *B. pilosicoli* was 4.36% in 46 farms in the area of Minas Gerais during a survey on intestinal pathogens [29]. The previous findings in the region reported a higher prevalence (45.5%) from a total of 22 confined “wean to finish” and fattening farms that were on the no feed medication category [11]. A recent study in Poland also reported that the prevalence of herds infected with *B. pilosicoli* was 13.7% and with *B. hyodysenteriae* was 18.9% [30]. However, the differences in diagnostic strategies may also influence the results.

The most prevalent species detected in our study were *B. innocens* and *B. murdochii*. *B. innocens* was isolated from 34% of the sampled farms, being the only species recovered from 19% of the total farms. These results are similar to those found in Denmark [19], where these species were found in 34.2% and 19%, respectively, also from herds with no clinical signs observed at the time of sampling. Another study also found those species as the most prevalent from animals with diarrhea [3], but other studies that collected sampled from pigs diarrhea found *B. hyodysenteriae* and *B. pilosicoli* as the most prevalent species [4].

As it has been mentioned above, the prevalence of *B. murdochii* was higher than that of *B. innocens* in this study; these results are consistent with previous studies where *B. murdochii* was the most prevalent among the weakly hemolytic *Brachyspira* species found in Austria [5].

Our findings show that more than one species of *Brachyspira* were found in nine farms, all the cases had in common the presence of *B. innocens*. In agreement with the previous studies [4,8,9], different species were simultaneously identified in a farm, but mixed *Brachyspira* species were found in only four fecal samples. It was previously proposed [1], that more than one species of *Brachyspira* could be found in a sample, of which some might not be pathogenic, so this makes the diagnosis of pathogenic *Brachyspira* more difficult.

Several studies associate the presence of *B. innocens* and *B. murdochii* with mid catarrhal diarrhea and colitis in pigs with the presence of bugs in intestinal tissue samples [4]. Such enteric scenario has been reproduced experimentally [2]. In this context, the high prevalence of *B. innocens* and *B. murdochii* found in our study, as in other studies, emphasizes the need for further studies to determine the pathogenic potential of these spirochetes in healthy and diarrheic pigs, as it has been suggested previously [3].

To the best of our knowledge, this is the first study of herd prevalence and identification of the different *Brachyspira* species in the central region of Argentina, where most of the important pig production farms are located. The combination of different diagnostic tests allowed the identification of a larger number of isolates and this approach will help us to conduct further studies on pathogenicity, genotyping, and antibiotic susceptibility of *Brachyspira* species in Argentina.

## Conclusion

The present study provides epidemiological data about herd prevalence of the different *Brachyspira* species in Argentina, showing that the prevalence figure seems to be higher than that reported in other countries.

## Authors’ Contributions

AC, JP, AA, and GZ designed the research work. AC, JP, MFL, and PC carried out the laboratory work. AC and PT analyzed the Bioinformatic data. AC and JP made the statistical analysis and drafted the manuscript. GDC, GZ, and EC helped in manuscript preparation. All authors have read and approved the final manuscript.
